# Environmental influences on induction of itching and scratching using immersive virtual reality

**DOI:** 10.1038/s41598-023-42322-8

**Published:** 2023-10-05

**Authors:** Emmy W. T. van de Burgt, Robbert van der Mijn, Sara Fabbri, Monicque M. Lorist

**Affiliations:** 1 Department of Experimental Psychology, University of Groningen, Groningen, The Netherlands; 2 Research School of Behavioural and Cognitive Neurosciences, University of Groningen, Groningen, The Netherlands; 3 University of Applied Sciences, Utrecht, The Netherlands

**Keywords:** Psychology, Signs and symptoms

## Abstract

Chronic itching is a serious and uncomfortable condition. The scratch response might result in a vicious cycle of alternating itching and scratching. To develop psychological interventions for people suffering from chronic itching and to break the vicious itch-scratching-itch cycle, it is important to elucidate which environmental factors trigger itch sensations. Virtual reality (VR) techniques provide a useful tool to examine specific content characteristics in a three-dimensional (3D VR) environment and their influences on itch sensations and scratching behaviour. This article describes two experiments in which we focused on the effects of environmental information on itching and scratching behaviour. Additionally, in the second experiment, we examined the influence of having a chronic skin condition on sensitivity to itch induction. We found evidence for the importance of the content of audio–visual materials for the effectiveness in inducing feelings of itch in the observers. In both experiments, we observed significantly higher levels of perceived itch in the itch-inducing conditions than in the control condition. Moreover, the results showed that elevated levels of perceived itch were associated with an increase in scratching behaviours, which was especially salient in the contagious itch condition, in which perceived itch was accompanied by a significant increase in the number of scratches. Experiment 2 additionally showed increased perceived itch levels in participants who reported having a chronic skin condition, reflecting higher sensitivity to itch-inducing audio–visual stimuli in this group than in participants without a chronic skin condition. Based on the results we concluded that directing attention towards itch- or scratch aspects of related information in the environment and to the consequences for one’s own skin are effective tools to induce itch sensations and scratching behaviour. This knowledge provides tools for developing novel strategies in advising and treating people suffering from chronic itching and breaking the vicious itch-scratching-itch cycle.

## Introduction

Chronic itching or pruritus is a serious and very uncomfortable condition that affects a significant portion of the general population: over one in five persons will, at some point in their lives, experience an episode of more than six weeks of itching^1^. Chronic itching has been associated with increased psychological stress, a negative body concept, and a decreased quality of life^2–6^. Itching is not only a very salient stimulus that automatically captures our attention^7^, it is also difficult to disengage attention from the itch sensation once attention is drawn to it, and shift attention to something else. Moreover, itch sensations serve as a natural impulse prompting individuals to scratch the affected area. While scratching can offer temporary relief by eliminating the irritants on the skin's surface, in more severe cases, this instinctive response can lead to harm to the skin's epithelial cells. The resultant epithelial stress response may trigger the release of cytokines, proteases, and AMPs (antimicrobial peptides) capable of activating immune cells and itch-sensory neurons. Subsequently, the activated immune cells foster inflammation, while neuropeptides released from neurons contribute to neurogenic inflammation. This inflammatory process induces itch, thus initiating a detrimental cycle of alternating itch sensations and scratching behaviour. Consequently, these mechanisms can exacerbate the symptoms significantly and contribute to the development of chronic itch pathology^8–11^.

To develop psychological interventions for people suffering from chronic itching and to break the vicious itch-scratching-itch cycle, it is important to elucidate which environmental factors trigger itch sensations^12^. Virtual reality (VR) techniques provide a useful tool to modulate our environment in a controlled and systematic way, allowing us to examine specific content characteristics in a three-dimensional (3D VR) environment and their influences on itch sensations and itching-related behaviour. With 3D VR, audio–visual stimuli can be presented in a realistic manner, where the audio–visual stimulus comes closest to reality and there are no distractions from other environmental stimuli. To the best of our knowledge, until this point in time, the VR technique has never been used to examine the influence of different environmental factors on the induction of itch sensations and scratching behaviour.

Besides studying the influence of environmental factors on itching and itch-related behaviour, the use of VR might also provide an alternative method to induce itching. In a research environment, the induction of itching by mechanical-, thermal-, chemical- (e.g., histamine) and electrical- stimulation of the skin is generally accepted^13^. However, these methods have drawbacks. Histamine-induced itch, for example, is invasive or causes skin manipulation in case of induction by histamine iontophoresis. This could be unpleasant for the participant. In addition, histamine-induced itch is experienced as more biting, painful, burning and piercing than audio visually-induced itch and lasts more than 15 min^14,15^. The induction of itching using VR environments instead, might have relevant advantages. It is non-invasive, non-skin manipulating, more comfortable and the participant is not distracted by other environmental stimuli.

Itching provoked by means of audio–visual content was first demonstrated by Niemeier et al.^16^, using a 2D environment. They showed itch-inducing pictures (e.g., fleas, mites, scratch marks on the skin, allergic reactions) to people who were interested in the scientific backgrounds of itch during an “itch-inducing lecture”. People in the audience indeed reported more itch, this effect was stronger in people who had a skin condition. In addition, Papoiu et al.^17^ investigated whether exposure to visual cues of itch could induce or intensify itch in healthy subjects and in patients with atopic dermatitis (AD). They showed a 5-min video clip of people scratching their left forearm and a control video clip (people sitting idle). Both groups reported increased itch intensity to the experimental video. However, the itch intensity and the number of scratch movements were significantly higher in AD-patients. Lloyd and colleagues^18^ were the first to take into account environmental factors to trigger itch sensations. They studied itch- and scratch responses in relation to specific itch-related visual information in healthy participants. In their study, the feeling of itch increased most in response to itch-related stimuli making direct contact with a person’s skin (e.g., ants crawling on the skin) or simply being present in the context (e.g., ants crawling on the ground). In contrast, scratch responses increased most in response to pictures of people actually showing scratching behaviour (e.g., scratching an insect bite). In the present study, we will use the immersive 3D VR environment to explicitly examine the influence of different environmental factors on the induction of itching and scratching behaviour.

**Figure 1:**
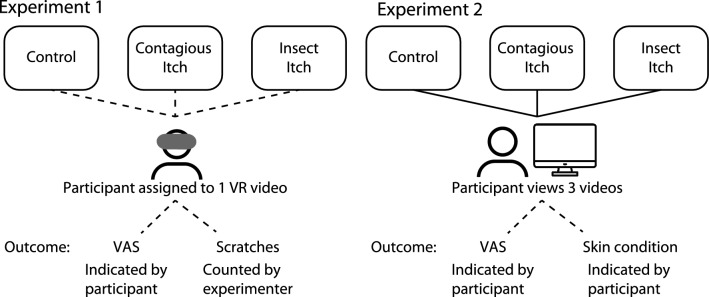
Graphic depiction of the study design.

This study describes two experiments (see Fig. 1) in which we focused on the effect of environmental information on itching and scratching behaviour. In the first experiment, we aimed to examine whether immersion in a virtual itch-inducing 3D VR environment is effective in inducing the sensation of itch and in inducing scratching behaviour in healthy participants. We especially focused on what environmental characteristics might have the strongest effects on both variables. During this experiment, healthy participants watched one of three 3D VR videos. Two videos (i.e., a *contagious* itch video and an *insect* video) were meant to be itch-inducing and a third video served as a neutral *control* video. In line with previous research^12,14–19^, we hypothesised that immersion of participants into one of the two itch-inducing 3D VR environments would evoke a stronger sensation of itch and more scratching behaviour than immersion into the neutral control environment. We also hypothesised that the subjective feelings of itchiness would increase participant’s scratching behaviour.

A second experiment was performed to investigate the influence of the content of the videos on itch induction more thoroughly and to examine the influence of having a chronic skin condition on sensitivity to itch induction. During the second experiment, participants watched 3 newly-made videos. As a result of Covid-19 restrictions, the experiment was carried out online utilising 2D videos. This approach enabled us to effectively address our inquiries. Again, two videos (i.e. a *contagious* itch video and an *insect* itch video) were meant to be itch-inducing and a third video served as a neutral *control* video. Based on the first experiment’s results and on previous research, the content of the videos was modified to optimise itch induction^14,18^.

## Experiment 1

### Materials and methods

#### Study design and participants

Sixty first-year psychology students (36 females; age *M* = 20.4, *SD* = 1.9) gave their written informed consent to participate in this experiment that was approved by the Ethics Committee of Psychology of the University of Groningen (code: PSY-1819-S-0136). They received course credits in exchange for their participation. The study was conducted in accordance with the Declaration of Helsinki. All participants had normal or corrected-to-normal vision. A between subjects, quantitative experimental design was used. Video was used as an independent categorical variable. The dependent variables were the number of scratches and the rating from the VAS scale.

### Materials

#### Self-Reported Itching Intensity visual analogue questionnaire

Itch intensity was measured using a visual analogue scale (VAS), using labels from 0 to 100, where zero reflects *no itch at all*, and 100 reflects the *worst itch imaginable*^20^. This questionnaire was administered digitally.

#### Behaviour recording

For recording the participants’ scratching behaviour, a digital camera (Olympus stylus SP-100EE) was used. To measure scratching behaviour, the video footages were examined and scratch frequency was counted in accordance with an established protocol by the experimenter [E.B.]. According to this protocol, scratching was defined as movement of participant’s fingertips across the same skin area for more than a second^18^.

#### Videos

Three 360° videos were filmed using the Ricoh Theta V 360° camera. The videos were presented using the Oculus Go VR-headset with a resolution of 2560 × 1440 pixels. Two videos were intended to induce itch (contagious itch video, insect itch video) and the third video served as a control video.

##### Contagious itch video

For the contagious itch video, the camera was put on a tripod and filmed six actors sitting in a circle in a seminar room, having a casual conversation about their housing situation while scratching themselves repeatedly on their body. To increase the feeling of presence in the virtual environment the camera was positioned to mimic the point of view of a seventh person in the conversation circle.

##### Insect itch video

For the video recording of the insect itch condition, a large (ca. 1 × 0.5 × 0.5 m) transparent box filled with sand and approximately 100 ants (Lasius Niger) were used. The camera was then positioned in the middle of the sand, so that the lens was just above the surface level of the sand. In the insect itch condition, the viewer of the video would feel surrounded by ants on the same level. The ants were not in contact with the skin of the participant or on the skin of another person.

##### Control video

The video for the control condition contained the same setting (same actors, room, viewpoint and conversation content) but without actors performing scratching behaviour. Each video had a duration of approximately 5 min. Stimulus material is available on request via Open Science Framework: https://osf.io/mv3q7/.

### Procedure

Participants were randomly allocated to one of the three videos (contagious itch, insect itch or control condition). Before starting the experiment, the participant read the research information and signed the informed consent. The participant was not informed about the actual research question beforehand, because research showed that even the mere act of thinking about itch can already induce itch^16^. Instead, the participant only was informed that the study would be about the ‘Effects of virtual reality on behaviour’. After signing the informed consent, the participant was seated on a rotating chair, allowing some freedom of movement while watching the VR video in order to increase the feeling of immersion into the virtual environment. The participant was asked to pay attention to the video, because the researcher would ask some questions about the content of the video afterwards. Prior to viewing, the Oculus Go was fitted to the participant’s head. Once the participant confirmed a satisfactory fit, the VR video and the video recordings were started. During the VR-exposure, the researcher stayed in the testing room silently to be able to react to possible technical difficulties. Right after the VR exposure, the participant was asked to fill in the VAS questionnaire in a different room. To conclude the experimental session, the participant received a debriefing form stating the actual purpose of the study and was thanked for his/her participation. In total, the session lasted around 20–25 min.

### Statistical analysis

Data were analysed in R4.2.1^21^. using the packages lme4^22^ and lmerTest^23^. A between-subjects, quantitative experimental design was used. To determine if immersion in the VR environments influenced scratching behaviour or perceived itch, two separate generalised linear effects models (GLM) were estimated. In both models, video was used as independent categorical variable. In one model the dependent variable was the number of scratches. In the other model the dependent variable was the rating on the VAS scale. GLM was used to accommodate the Poisson distribution of the dependent variables. To determine if the effect of the video was significant, the dependent variables were added to intercept-only models. Bayes factors (denoted as BF_01_) were estimated by comparing Bayes Information Criterion (BIC) of intercept-only models (denoted as M_0_) to the resulting models (denoted as M_1_)^24^. Resulting BFs between 3 and 20 are considered positive evidence in favour of M_1_, BFs between 20 and 150 are considered strong evidence, and BFs over 150 are considered very strong evidence^25^. Three post hoc comparisons (Control vs. Insect, Control vs. Contagious, and Insect vs. Contagious) were made by separately estimating BFs using only the data from the corresponding videos. A similar approach was followed to determine the influence of experienced itch on scratching. BF was estimated by comparing an intercept-only model containing scratch count as dependent factor (M_0_) to a model that included VAS as independent factor (M_1_). All analysis scripts are made available via Open Science Framework: https://osf.io/mv3q7/.

## Results

### Self-reported itching intensity (VAS)

**Figure 2:**
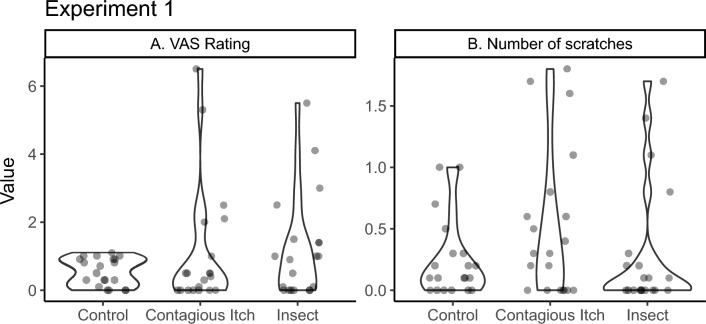
Effects of video on VAS-scores and number of scratches. Violin plots indicate the distribution of observed responses. Each dot represents the mean value of a single participant.

The distribution of the VAS scores (self-reported itch intensity, Fig. 2 a) was heavily right-skewed (Mean = 9.52; SD = 13.93; N = 60). Feelings of itch were higher in the contagious itch video (Mean = 11.10; SD = 18.15) and insect itch video (Mean = 12.00; SD = 15.18) conditions as compared to the control video (Mean = 5.45; SD = 4.03) condition, as presented in Fig. 2 a. There was very strong evidence in favour of the model including video (M_1_) over the intercept-only model M_0_ (BF > 1000). The final models’ estimates are shown in Table 1. All levels of the experimental conditions were compared to each other by separately estimating BFs using only the data of the compared videos. We found very strong evidence that perceived itch (VAS) was higher in the contagious itch group (BF > 1000) and insect itch group (BF > 1000) compared to the control group. There was positive evidence against a difference between the contagious itch group and the insect itch group (*BF* = 0.225).

**Table 1:** Estimated model means.

	VAS		Scratching	
Video	Estimate	95% conf.int	Estimate	95% conf.int
Control	5.45	[4.52, 6.58]	2.45	[1.85, 3.24]
Contagious Itch	11.10	[9.73, 12.66]	5.05	[4.16, 6.14]
Insect	12.00	[10.57, 13.62]	3.00	[2.33, 3.86]

### Observed scratching frequency

The distribution of the scratching frequencies (Fig. 2 b) was also heavily right-skewed (Mean = 3.50; SD = 4.97; N = 60). Twenty-three of the 60 participants did not scratch at all. In the other participants, the scratching frequency varied between one and eighteen. The number of scratches was highest in the contagious itch video (Mean = 5.05; SD = 6.00) compared to the insect itch video (Mean = 3.00; SD = 5.18) and the control video condition (Mean = 2.45; SD = 3.15). There was strong evidence in favour of the model including video over the intercept-only model (BF > 1000). The parameter estimates for the coefficients are shown in Table 1. All experimental conditions were compared to each other by separately estimating BFs using only the data of the compared videos. We found strong evidence that the number of scratches was higher in the contagious itch group compared to the insect itch group (BF = 30.0) and the control group (BF > 1000, very strong evidence). There was positive evidence against a difference between the control group and the insect itch group (*BFp* = 0.276).

Finally, elevated levels of perceived itch led participants to perform more scratching behaviours. A model predicting scratching frequency with VAS as a fixed factor, M_1_, was more favourable than the intercept-only model M_0_ (BF > 1000, indicating very strong evidence). The positive parameter estimate of VAS indicated the positive relationship. We concluded that both scratching and perceived itch are influenced by the type of video. Scratch frequency is mostly affected by the contagious itch video, while the insect video does not significantly differ from the control. For perceived itch (VAS) both conditions show differences, but separate BF analysis reveals no difference between contagious itch and insect videos.

## Experiment 2

A second experiment was performed to investigate the influence of the content of the videos on itch induction more thoroughly and to examine the influence of having a chronic skin condition on sensitivity to itch induction. During this experiment, participants watched three newly made 2D videos accompanied with an online survey. The decision to employ 2D videos was a pragmatic solution in light of the prevailing Covid-19 restrictions. However, due to our emphasis on the video content, the inclusion of 2D videos within an online survey proved adequate for the purposes of this experiment. Based on the results of the first experiment and on previous research, the content of the videos was modified to optimise itch induction^14,18,26^. In the social contagious video, participants’ attention was focused more on the actors’ scratching by having them talk about the scratching along with the scratching itself^14,26^. In the insect video, the same group members show photo and video footage of insects on the skin and their consequences for the skin, while talking about the insects, but not scratching^18^. Additionally, we included a question for indicating whether a participant was at the moment of participating in the study having any chronic skin conditions.

## Materials and methods

### Study design and participants

One hundred five participants gave their written informed consent to participate in this experiment that was approved by the Ethics Committee of Psychology of the University of Groningen (code: PSY-1920-S-0346). The participants were recruited by means of convenience sampling either through the participant pool of the University of Groningen or via various social media platforms, which targeted both students and non-students. The participant pool is an online platform used by undergraduate students to collect credits by participating in studies. The participants, recruited through various social media platforms, participated without receiving any compensation. The study was conducted in accordance with the Declaration of Helsinki. All participants indicated to have normal or corrected-to-normal vision. Exclusion criteria were solely incomplete survey responses. Thirty participants had to be excluded due to incomplete completion of the online survey. Seventy-five participants completed the experiment (54 females; age M = 22, SD = 3.8). Ninety-two percent of the participants were students. A within-subjects quantitative experimental design was used which allowed us to expose each participant to all video conditions. Again, video was used as an independent categorical variable. The dependent variables were the rating from the VAS scale, and whether the participant indicated the presence of a skin condition.

### Materials

#### Self-reported itching intensity visual analogue questionnaire

Itch intensity was measured using a horizontal version of the VAS, on which the amount of itch was self-rated on a continuum, ranging from 0.0 (*no itch*) to 10.0 (*extreme itch*)^20^. VAS scores were linearly transformed to match the 0–100 scale of Experiment 1.

#### Other questionnaires

Additionally, we included a question for indicating whether a participant was at the moment of participating in the study having any chronic skin conditions. Exploratively, we included a number of questionnaires: To measure participants’ level of mindfulness, the Mindful Attention Awareness Scale (MAAS)^27^ was used. The State-Trait Anxiety Inventory (STAI)^28^ was used to measure the level of anxiety. The level of skin sensitivity during the past three days was measured on the Sensitive scale-14 (SS-14)^29^. The data of the exploratively included questionnaires are not described in this paper. All questionnaires were administered digitally.

#### Videos

Three 360° videos were filmed using the Ricoh Theta V 360° camera. The first two videos were intended to induce itching (the contagious itch video and the insect itch video) and the third video served as a control video. Each video lasted approximately three minutes and presented a group of three students sitting at the table while having a conversation. The angle of view in the videos was in the perspective of a fourth person sitting at the table to increase the engagement with the group setting.

##### Contagious itch video

To measure the effect of socially contagious itch, participants were exposed to scratching carried out by the group members presented in the video while listening to their itch-related discussion. The conversation concerns a rash on the right arm of one of the group members. Over the course of the conversation the group tries to find out what may have caused the rash. The person with the rash continuously scratches herself and shows the rash to the participant. While talking, the other two conversational partners start to scratch as well. While the person with the rash scratches herself very explicitly, the other group members scratch in a less salient way.

##### Insect itch video

The insect itch video consisted of the same group members who presented various kinds of photo and video footage of itch-related stimuli. The participant is shown a video of a bedbug and pictures of reddened skin that was caused by their bites. Next, the conversation shifts to other parasites that are associated with itch. First to mosquitos (a picture of an infected hand caused by mosquito bites is shown to the participant) and then to scabies (the participant is shown a graphical illustration of *sarcoptes scabiei*, the mites who cause the skin condition scabies, building tunnels under human skin). All the topics discussed concerned itch-inducing parasite infections. The topic of the conversation comprised of discussing the footage shown and no scratching was present.

##### Control video

In this video, the conversation was coordinated to be neutral and thus not itch inducing. The same group member that had the rash in the contagious itch video, now has a bruise drawn on her arm. She talks about the bruise being very painful and shows the bruise to the camera. She tells the others that she fell down the stairs in her student house. The topic of this conversation is traditional Dutch houses and what dangers their architectural style entails. In the video, participants do not scratch and do not discuss any itch-related topics. Stimulus material is available on request via Open Science Framework: https://osf.io/mv3q7/

### Procedure

The participants received a link to the experiment either through their university’s SONA account or through their social media platforms. The experiment was completed via the Qualtrics online survey software^30^. Because we used a survey, the 360° (3D) videos were shown in 2D in the browser of the participant. The experiment started by informing the participant about the study. In particular, they were informed about the actual research question (mentioning itch) beforehand. After giving their informed consent, the participants were asked to fill out the three questionnaires. The participants were asked to indicate whether they knowingly have a skin condition (i.e., ‘yes’ or ‘no’). Then, they were presented with the three videos in a randomised order. After each video, participants were asked to rate their level of experienced itchiness on a VAS scale from 0.0 to 10.0. Subsequently, the debriefing was presented, including the information that no deception was used in this study.

### Statistical analysis

In Experiment 2 a similar analysis was used as for Experiment 1. To determine if the videos influenced perceived itch, and if these effects were different for participants with or without a skin condition, generalised linear mixed effects regression (GLMER) models were estimated. The dependent variables were the rating from the VAS scale. Video and whether the participant indicated the presence of a skin condition were used as independent categorical variables. Participant was added as random effect. Six post hoc comparisons (Control vs. Insect, Control vs. Contagious, and Insect vs. Contagious) were made by separately estimating BFs using only the data from the corresponding videos. This was done for both the participants with and without a skin condition.

## Results

**Figure 3:**
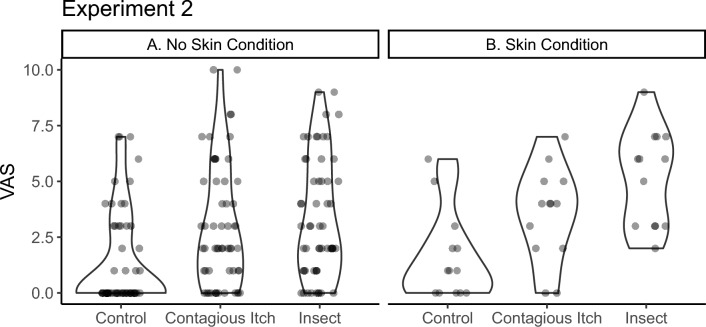
Violin-plot showing the Effect of the Videos on VAS separately for the participants with no skin condition (**A**) and with skin condition (**B**).

The distribution of the VAS-scores (self-reported itch intensity, Fig. 3 ) was heavily right-skewed. Feelings of itch were higher in the contagious itch video (Mean = 30.7; SD = 26.2) and insect itch video (Mean = 36.9; SD = 26.5) conditions as compared to the control video (Mean = 14.3; SD = 20.9), as presented in Fig. 3 a. There was very strong evidence in favour of the model including video (M_1_) over the intercept-only model M_0_ (BF > 1000). There was strong evidence against adding skin condition (M_1_) to the intercept-only model M_0_ (BF > 1000). However, adding skin condition as an interaction with video was favourable over the video-only model (BF = 13.1, positive evidence). The final models’ estimates are shown in Table 2. All levels of the experimental conditions were compared to each other by separately estimating BFs using only the data of the compared levels. We found that perceived itch (VAS) was higher for the contagious itch video (BF > 1000, very strong evidence) and insect itch video (BF > 1000, very strong evidence) compared to the control video for participants with no skin condition. There was also a difference between the contagious itch video and the insect itch video (BF = 78, strong evidence). In the participants with a skin condition, perceived itch was higher for the insect video compared to the control video (BF > 1000, strong evidence) and the contagious itch video (BF > 1000). Perceived itch was also higher for the contagious itch video compared to the control video (BF > 1000).

**Table 2:** Estimated model means.

	Skin condition		No skin condition	
Video	VAS Estimate	95% conf.int	VAS Estimate	95% conf.int
Control	14.3	[7.6, 26.8]	8.3	[6.1, 11.3]
Contagious Itch	31.4	[16.9, 58.3]	17.8	[13.2, 24.1]
Insect	45.7	[24.6, 84.8]	20.1	[14.1, 27.2]

## Discussion

In this article, we described two experiments in which we focused on the effect of environmental information on itching and scratching behaviour. In the first experiment, we aimed to examine whether immersion in a virtual itch-inducing 3D VR environment is effective in inducing the sensation of itching and in inducing scratching behaviour in healthy participants and to examine which environment is more capable of inducing itching and scratching behaviour. Based on the results of the first experiment, the aim of the second experiment was to investigate the influence of the content of the videos on itch induction more thoroughly and to examine the influence of having a chronic skin condition on sensitivity to itch induction by these environments.

In both experiments, we used two videos that were designed to elicit itching through distinct itch-related stimuli: a contagious itch video and an insect itch video. Additionally, in both experiments, the third video served as a neutral control video.

In Experiment 1, we demonstrated that immersion in a virtual 3D itch-inducing environment is effective in inducing the sensation of itching and scratching behaviour in healthy participants. This is in line with previous research, but up until now, the 3D VR was never used to establish this effect^12,15–19^. There was no difference between the contagious itch group and the insect itch group in terms of perceived itch (VAS). However, we found that scratch frequency was higher in the contagious itch group compared to the insect itch group and the control group. This implies that observing someone scratching is a potent stimulus for initiating the act of scratching. These findings are consistent with previous studies^15,17,18^. Furthermore, elevated levels of perceived itching led participants to perform more scratching behaviours, which is according to our hypothesis.

During Experiment 2, participants watched three newly designed 2D videos accompanied with an online survey. Based on the results of the first experiment and on previous research^14,26^, the content of the videos was modified to optimise itch induction. In the social contagious video, we directed participants’ attention towards the actors’ scratching behaviour by not only depicting them scratching themselves as in the video used in the first experiment, but also incorporating verbal discussions about the act of scratching alongside the observable behaviour^14,26^. In the insect video, the participants were shown photo and video footage of insects situated directly on the skin and its consequences for the skin (e.g. scratching effects, redness), while the actors were talking about the insects, but did not show scratching behaviour^18^. Sensations of itch, as measured by the scores on the visual analogue scale (VAS), exhibited higher intensity in both the contagious itch and the insect itch conditions when compared to the control condition. Notably, this effect seemed more pronounced among participants who reported having a chronic skin condition than those who did not.

In line with previous research, our findings confirm that environmental information (and not exclusively physical stimuli) can induce itching and scratching behaviour^14–19^. In addition, we found preliminary evidence for the importance of the content of audio–visual materials for the effectiveness in inducing feelings of itch. The more explicit focus of participants’ attention on actors’ scratching by having them, for example, talk about the scratching along with the scratching itself in the social contagious video, seemed more effective in itch induction (as evidenced by higher VAS scores) than only seeing people scratching. In the insect video, using photo and video footage of insects on the skin and their consequences for the skin, while directing the focus on the topic by talking about the insects in the insect video, seemed more effective in itching induction (as evidenced by higher VAS scores) than feeling surrounded by insects not in direct contact with the skin.

In Experiment 2, due to Covid-19 restrictions, we demonstrated these effects through the presentation of the videos in a 2D format within an online survey. Consequently, the results of the two experiments are not fully comparable. It would therefore be highly valuable to replicate Experiment 2 within a laboratory setting, utilising 3D VR videos. Considering that 3D VR provides an audio–visual experience that closely mimics reality and effectively eliminates potential distractions from external stimuli, we anticipate a further increased perception of sensations of itching. Furthermore, in contrast to Experiment 1, in Experiment 2, the participants received explicit information regarding the primary research question (pertaining to itching) through the provided instructions. This emphasis on itching might have indirectly guided their attention towards the sensation of itching, potentially impacting the resultant VAS scores in Experiment 2^31^. To gain more insight into factors that play a role in inducing feelings of itch and scratching behaviour in people with a chronic skin condition , in Experiment 2, the participants were asked to indicate whether they knowingly had a chronic skin condition (responding with either ‘yes’ or ‘no’). We found that participants with chronic skin conditions are more sensitive to itch-inducing audio–visual stimuli than participants without a chronic skin condition. This is in line with research by Papoiu et al.^17^ and Lloyd et al.^18^. Peripheral and central sensitization in people with a chronic skin condition could contribute to this^13^ just as attentional bias towards itch-related words^7^ and attentional focus on bodily sensations^26^.

Our findings hold potential value for the development of novel strategies in advising and treating people suffering from chronic itching for several reasons. First, with an enhanced understanding of the environments that intensify itching, you gain the means to circumvent conditions that trigger itch. For example, our results indicate that in the hospital it is better not to have two patients with chronic itching in the same room. They could worsen each other's conditions due to, for example, exposure to contagious scratching behaviour^12^. Additionally, based on the hypothesis that classical conditioning is a possible mechanism underlying the induction of itch by audio–visual stimuli, it might be possible to extinct the conditioned behaviour (i.e. scratching) through pairing the itch-inducing stimuli with an itch alleviating stimulus in a controlled environment using the VR technique^12^.

Furthermore, knowledge about what content is most itch-inducing could be applicable in designing VR environments that can be used as an alternative method to induce itching for research^14^. For example, itch induction by immersive 3D VR environments can be used for research into an itch-reducing intervention. The replacement of methods generally used to induce itching (e.g., mechanical-, thermal-, chemical- (e.g., histamine) and electrical- stimulation) by induction of itching using VR environments has relevant advantages. It is non-invasive, more comfortable for the participant, and in particular, it allows us to show moment-to-moment fluctuations in subjective itchiness in relation to an itch reducing intervention.

In Experiment 2, we found preliminary evidence for the importance of the content of audio–visual materials for the effectiveness in inducing feelings of itch. The more explicit focus of participants’ attention on actors’ scratching seemed more effective in itch induction (as evidenced by higher VAS scores) than only seeing people scratching. Van Laarhoven et al. (2010) also found that a greater attentional focus on bodily sensations is relevant to the experience of pain and itch sensations. Diverting one’s focus away from the pain can potentially alleviate the sensation. Immersive 3D VR environments have been shown to reduce pain^32^. Since parallels exist in the perception of both pain and itch^13,33^, it would be beneficial to explore the potential of immersive 3D VR environments in mitigating the sensation of itching. Such exploration could extend to breaking the potentially vicious cycle of itch-scratching-itch.

## Data Availability

The data are available from the corresponding author (E.B.) upon reasonable request. Analysis scripts are available at https://osf.io/mv3q7/ .

## Abbreviations

AD: Atopic dermatitis
AMPs: Antimicrobial peptides
BF: Bayes factors
GLM: Generalized linear effects model
GLMER: Generalized linear mixed effects regression
M: Mean
MAD: Median absolute deviation
MAAS: Mindful attention awareness scale
SD: Standard deviation
SS-14: Sensitive scale-14
STAI: State-trait anxiety inventory
VR: Virtual reality
2D: Two dimensional
3D VR: Three dimensional virtual reality
VAS: Visual analogue scale
